# Expression Signature of the AT-Rich Interactive Domain Gene Family Identified in Digestive Cancer

**DOI:** 10.3389/fmed.2021.775357

**Published:** 2022-01-20

**Authors:** Yongqu Lu, Zhenzhen Liu, Wendong Wang, Xin Chen, Xin Zhou, Wei Fu

**Affiliations:** Department of General Surgery, Peking University Third Hospital, Beijing, China

**Keywords:** AT-rich interactive domain, digestive cancer, gene family, immune subtype, tumour microenvironment

## Abstract

**Background:**

The AT-rich interactive domain (ARID) gene family of 15 proteins has an important role in development and proliferation. Gene expression alterations of the ARID family are correlated with the pathogenesis of digestive cancer, but systematic research has not been conducted.

**Methods:**

We obtained transcriptome sequencing data, clinical characteristics and stemness indices of the seven main types of digestive cancer (cholangiocarcinoma, colon adenocarcinoma, oesophageal carcinoma, liver hepatocellular carcinoma, pancreatic adenocarcinoma, rectum adenocarcinoma and stomach adenocarcinoma) from public pan-cancer data to combine the analysis of the expression and prognostic signature of the ARID gene family. The stromal and immune scores for each sample were calculated to explore the correlations between the ARID gene family members and the tumour microenvironment.

**Results:**

After screening, 1,920 digestive cancer samples were included in our study. *ARID3C* was expressed at low levels throughout the digestive cancer samples. The expression levels of *ARID1A* and *JARID1C* were relatively high, but there was striking heterogeneity across the different cancer types for specific family members. The survival analysis indicated that many genes were significantly related to the prognosis of patients with liver hepatocellular carcinoma. The stemness indices, stromal score, and immune score analysis showed that the expression of a single ARID gene had characteristic consistency in each tumour, but the levels among the different genes still varied.

**Conclusion:**

Our systematic study of the ARID gene family and its association with the immune infiltrate, tumour microenvironment and outcomes of digestive cancer patients focus on the complex relations and indicate the need to study each ARID member as an individual in a specific cancer type.

## Introduction

Proteins with AT-rich interactive domains (ARIDs), which are helix–turn–helix motif-based DNA-binding domains spanning ~100 amino acid residues, constitute a highly conserved family. Interestingly, not all ARID-containing proteins conform to a specific pattern of binding sequences and AT-rich sequences (1). However, ARID family proteins still participate in a comprehensive range of DNA interactions (2). In humans, 15 members have been identified. They fall into seven classes based on the degree of sequence identity, and these classes are named ARID1 through ARID5, JARID1 and JARID2 in mammals (3, 4). Members of the ARID family are involved in the modification of chromatin structure and regulate the expression of specific genes that are directly associated with cellular growth, differentiation, and development (5, 6). The precise functions of all the ARID family proteins are still not entirely clear.

Digestive organs, including the liver, gallbladder, pancreas, oesophagus, stomach and intestine, provide suitable conditions for the transport and absorption of food and nutrient metabolism. These digestive organs are impacted by our diet to a great extent, and they are easily susceptible to various diseases with obvious symptoms. Patients with solid tumours that originate from these organs tend to have higher morbidity and mortality compared with other organ systems (7). Previous studies have shown that there are common intersections between tumorigenesis mechanisms and regulatory networks in different digestive cancers (8–10).

The ARID gene family has been confirmed to participate in tumour growth and invasion. Downregulation of *ARID1A* and *ARID1B*, the variant subunits of the BRG1/BRM-associated factor complex from the SWI/SNF chromatin remodelling family, in various cancer cells might lead to deficiency in DNA repair (11). Another chromatin remodelling factor, *ARID2*, plays a metastasis suppressor role in hepatocellular carcinoma and rectal cancer, and mutants of this gene exhibit tumorigenic functions (12, 13). *ARID3A* promotes cell proliferation, migration and invasion in colorectal cancer and is significantly associated with the prognosis of patients (14). The mechanisms by which ARID family members function in different types of cancer often vary and even have contrary outcomes. *JARID2* has been reported to facilitate tumorigenesis and epithelial-mesenchymal transition in bladder cancer and acts as a tumour suppressor in myeloid neoplasms (15, 16). The close connections among family members and the certain similarities in the tumorigenesis and development of digestive cancer are the basis for the use of systematic analysis. Stem cell-like features effectively work in tumour initiation and are closely related to resistance to drug treatment. Tumour microenvironment refers to the initiation, development and metastasis that are closely related to the internal and external environment of tumour cells more than just the location. Stemness and tumour microenvironment can be used as auxiliary indicators reflected the status of cancer.

In the present study, we investigated the expression pattern of the ARID gene family members and their association with the stemness, the tumour microenvironment and patient outcomes in digestive cancer. Our study highlights the essential differences in the ARID genes that exist between digestive cancers and the need to study each ARID as a separate entity.

## Materials and Methods

### Data Preparation

The expression profiles, including the RNA sequencing data, and the corresponding clinical data, including tumour-node-metastasis (TNM) stage, stemness scores based on mRNA (RNAss), stemness scores based on DNA methylation (DNAss), and immune subtypes of cholangiocarcinoma (CHOL), colon adenocarcinoma (COAD), oesophageal carcinoma (ESCA), liver hepatocellular carcinoma (LIHC), pancreatic adenocarcinoma (PAAD), rectum adenocarcinoma (READ) and stomach adenocarcinoma (STAD) patients, were downloaded from the Xena platform (http://xena.ucsc.edu), which integrates cancer genomic and clinical resources (17). We selected the public database from The Cancer Genome Atlas (TCGA) for this study (18). The enrolled patients had a definite diagnosis of digestive cancer, and patients with an overall survival (OS) longer than 30 days were included for clinical analysis. RNA sequencing data were calibrated and log2 transformed. For pantumour analyses, gene expression was normalised to housekeeping genes. In total, 1,920 samples were available for expression analysis, and the number of samples available for each cancer type ranged from 45 for CHOL to 512 for COAD (Supplementary Table 1). Among them, PAAD had fewer than five associated normal tissue samples, so only the remaining six cancer types were used to investigate the alterations of gene expression between tumours and normal tissues with a linear mixed effects model.

### Expression Analysis

Expression values of the ARID family were compared between the normal tissues and tumour samples with the limma package. Fold change was calculated and performed with log2 transformations. Correlation analysis between gene expression and the clinical parameters was performed to explore the associations of the family of genes with other characteristics using the same package. Protein expression levels were verified with immunohistochemistry from the Human Protein Atlas (HPA, https://www.proteinatlas.org) (19). Antibodies against *ARID1A, ARID1B, ARID5A*, and *ARID5B* were applied to HPA005456, HPA016511, HPA023879 and HPA015037, respectively.

### Survival Analysis

Patients were divided into a high-expression group and a low-expression group, and the cut-off was determined by the median level of expression for a single ARID member. Survival analysis was conducted with the survival package based on OS data.

### Tumour Cell Stemness and Microenvironment Analysis

Tumour stemness indices were extracted from transcriptomic and epigenetic data from the study of Malta et al. and were used to measure stem cell-like features (20). The Estimation of STromal and Immune cells in MAlignant Tumours using Expression data (ESTIMATE) is an interpretation that uses gene expression signatures to infer the infiltration levels of stromal and immune cells in tumours (21). The estimate package was used to calculate the scores that described the tumour constituents but not accurate purity (22). Six immune subtypes, wound healing (C1), interferon-γ (IFN-γ) dominant (C2), inflammatory (C3), lymphocyte depleted (C4), immunologically quiet (C5) and transforming growth factor-β (TGF-β) dominant (C6), were identified by immunooncologic gene signatures concluded from TCGA data, and these subtypes were used to clarify the association between ARID expression and immune infiltration (23). The Corrplot package was used to test the correlation between gene expression and stemness scores, estimate scores and immune subtypes.

### Statistical Analysis

All statistical analyses were performed in R (version 4.0.2). Wilcoxon and Kruskal–Wallis tests were used in differential analyses. Univariable Cox proportional hazards regression was used to estimate the hazard ratios (HRs), and the confidence interval (CI) was set at 95%. The survival curve was generated by the Kaplan–Meier method, and OS differences were evaluated using the log-rank test with the median value as the cut-off. Spearman and Pearson tests were conducted for correlation analyses. A *P* value < 0.05 was considered a significant difference in the statistical analyses.

## Results

### ARID Gene Family Expression in Digestive Cancer

**Figure 1:**
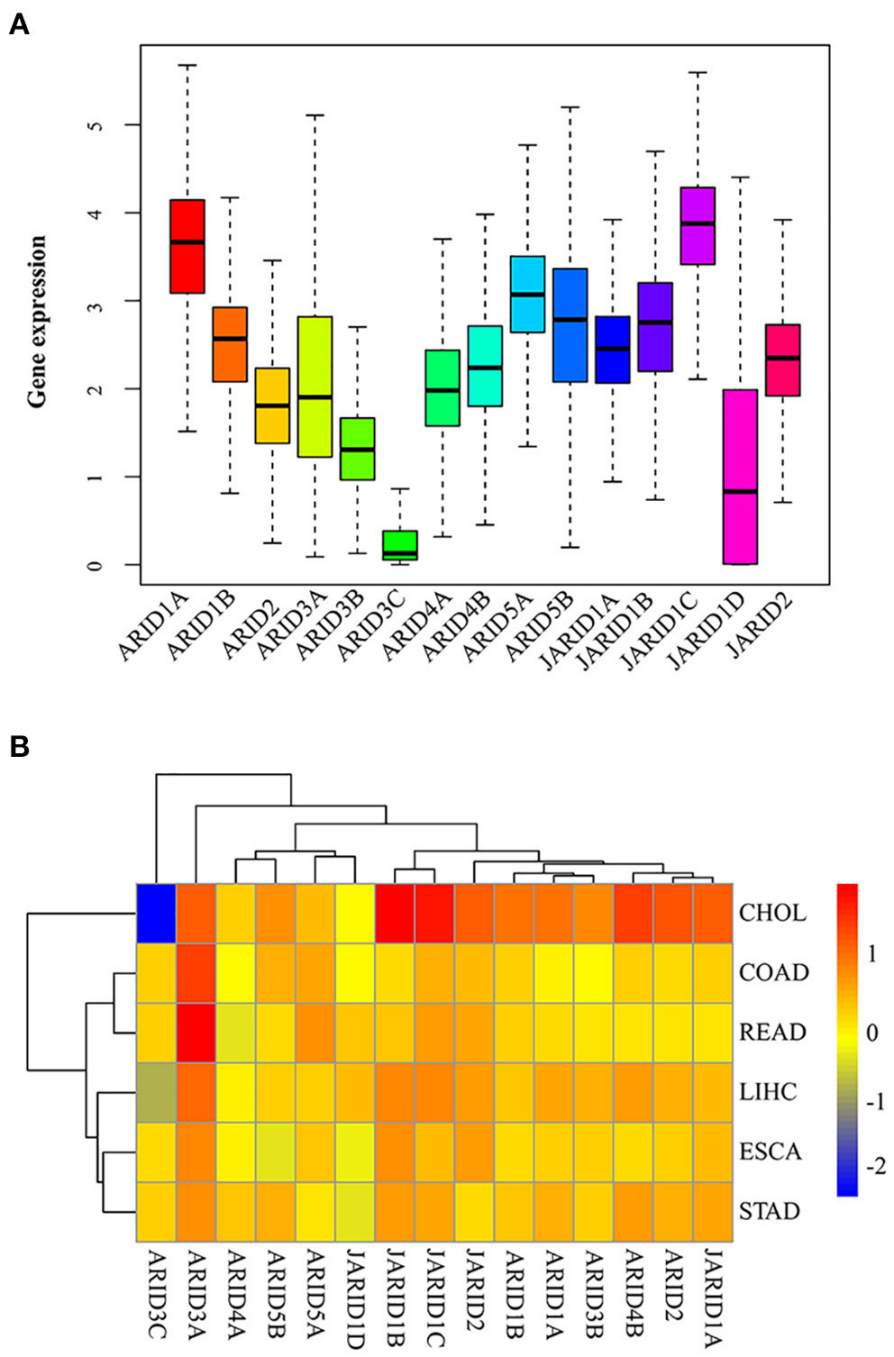
Expression levels of ARID gene family in digestive cancer. **(A)** Total expression of ARID family members across seven digestive cancer types. **(B)** Difference of ARID gene expression comparing tumour to normal tissues based on fold change of log2 transformation for six cancer types.

To better understand the intrinsic expression pattern of the ARID genes, we explored the expression levels of the ARID family members in seven digestive cancer types that were available in the TCGA RNA sequencing profile. Interior and exterior heterogeneity among the different digestive cancers regarding the expression levels of the corresponding genes were clearly observed in all 15 ARID members (Figure 1A). The expression of *ARID3C* was at a low level overall. *ARID1A* and *JARID1C* were the predominantly expressed genes in digestive cancer. In terms of a specific ARID family member, there was striking heterogeneity across different cancer types. There was a considerable difference in gene expression between tumour and normal tissues in some cancer types, which was in complete contrast to the lack of comparative significance in other cancer types (Figure 1B). For example, most ARID gene family members were highly expressed in CHOL, except for *ARID3C* and *JARID1D*. However, the differential expression of *ARID3C* in CHOL between tumour and normal tissues was more obvious than that in ESCA. *ARID3A* was prevalent high-expressed in tumour tissues of digestive cancers. The intrinsic differences in the expression of the ARID gene family members in different cancer types showed the need to integrate individual gene members for further analysis.

### Association of ARID Gene Expression With Patient Survival

**Figure 2:**
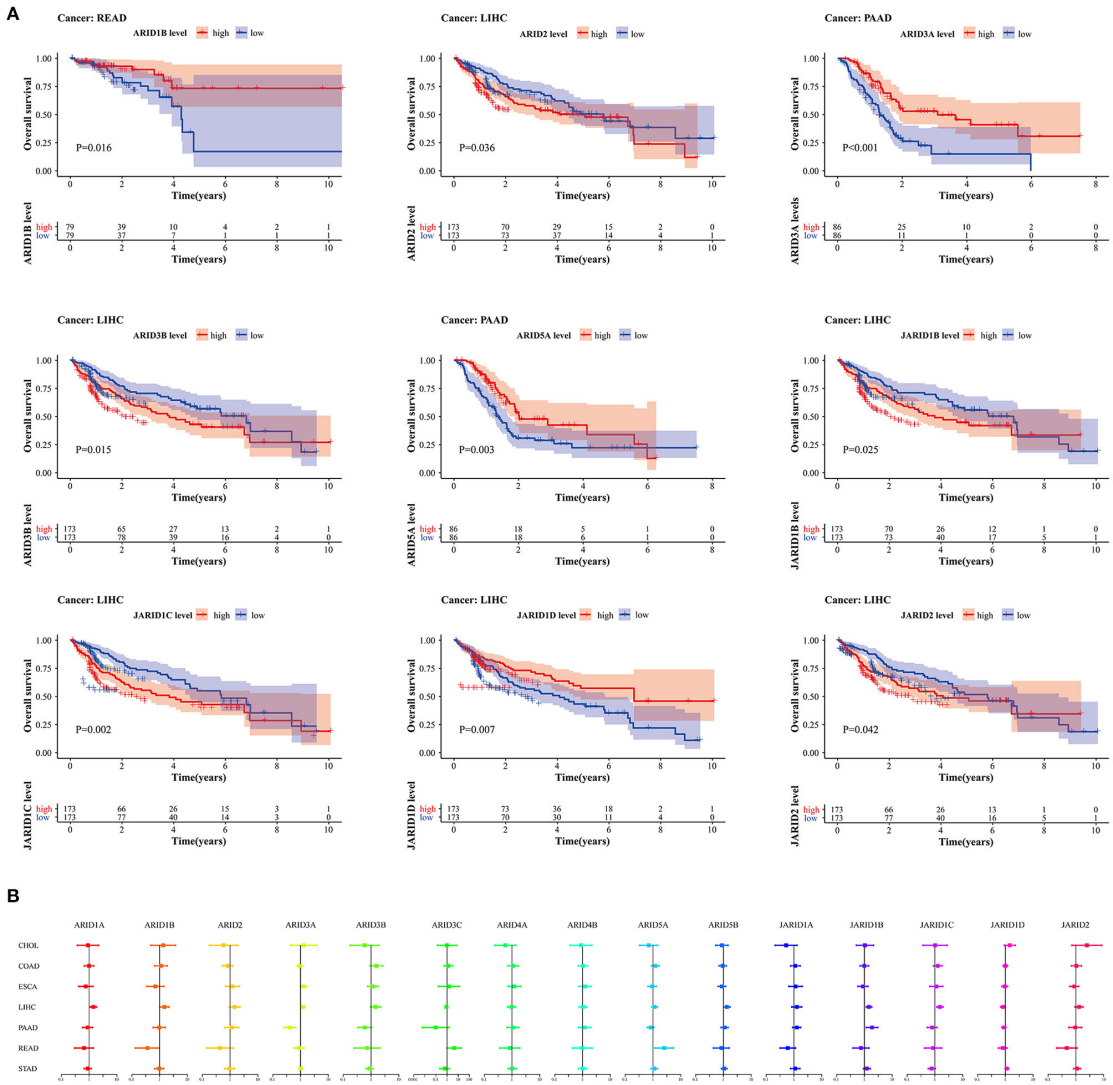
Prognostic significance for ARID family members in digestive cancer. **(A)** Survival curves with statistical significance for 15 ARIDs in different types of digestive cancer (*P* < 0.05). **(B)** Univariate Cox proportional hazard regression models show HRs and 95% CIs of OS for survival benefit and risk with increased expression of ARID family in different cancer types.

To correlate and accurately assess the prognostic value of the ARID family members in digestive cancer, clinical data from seven digestive cancers were used to investigate the relationship between ARID expression and patient overall survival. From the survival analysis, we found that *ARID2, ARID3B, JARID1B, JARID1C, JARID1D* and *JARID2* were generally relevant to the overall survival of LIHC patients. High-expressed *ARID3B* indicated worse prognosis in READ patients and High-expressed *ARID3A* and *ARID5A* suggested worse prognosis in PAAD patients (Figure 2A). However, the associations varied according to the ARID members and different cancer types. For example, the high expression of *ARID3A* was highly associated with the worse prognosis of PAAD patients, but there were no significant associations between this gene and prognosis in the other digestive cancers. The increased expression of *ARID3B* predicted a survival advantage in LIHC. The univariable Cox proportional hazard regression showed consistent results with the survival analyses. The prognostic value was gene specific for digestive cancers (Figure 2B; Supplementary Table 2).

### ARIDs Were Associated With the Immune Response in Digestive Cancers

**Figure 3:**
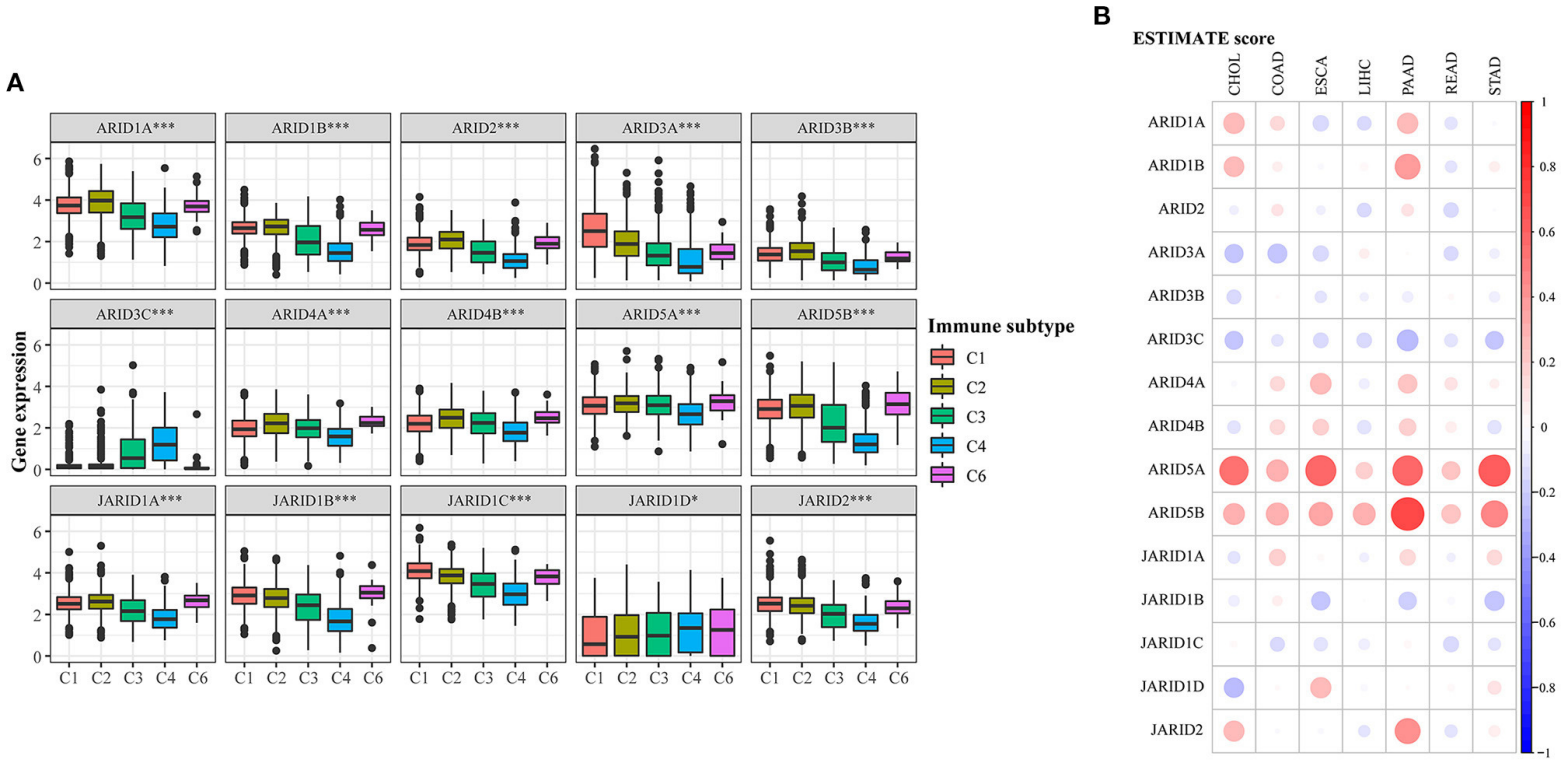
Association of ARIDs expressions with tumour microenvironment factors. **(A)** Association of ARIDs expression with immune infiltrate subtypes across digestive cancer types. *P < 0.05, **P < 0.01, ***P < 0.001. **(B)** Correlations between ARIDs expressions and ESTIMATE scores of digestive cancer. Bigger dot stands for higher correlation coefficient.

The microenvironment has a significant influence on tumour progression, and the response from immune cells is critically involved. Six types of immune infiltrates from C1 to C6 were featured in digestive cancer, and the expression of ARID family members was correlated with the subtypes in varying degrees (Figure 3A). No patient belonged to C5 subtype. *JARID1C* was overexpressed in all subtypes, while *ARID3C* was highly expressed in C3 and C4, suggested the close relationship with inflammation and lymphocyte depletion in microenvironment. Correlations between *ARID5B* and C6 infiltrates indicated that digestive tumours expressing *ARID5B* tended to be enriched with TGF-β. For a deep investigation of the relationship between ARID gene family members and the immune microenvironment in digestive cancer, we performed the ESTIMATE algorithm to evaluate the stromal and immune status in different samples. The results suggested that ARID family members and the ESTIMATE score for digestive cancer had a wide range of correlations (Figure 3B; Supplementary Table 3). *ARID5A* was highly associated with stromal and immune scores in CHOL, ESCA, PAAD and STAD. *ARID5B* had significant positive correlations with stromal and immune scores in PAAD, revealing that this gene might effectively function in the tumour microenvironment.

### ARIDs Were Associated With Stemness in Digestive Cancer

**Figure 4:**
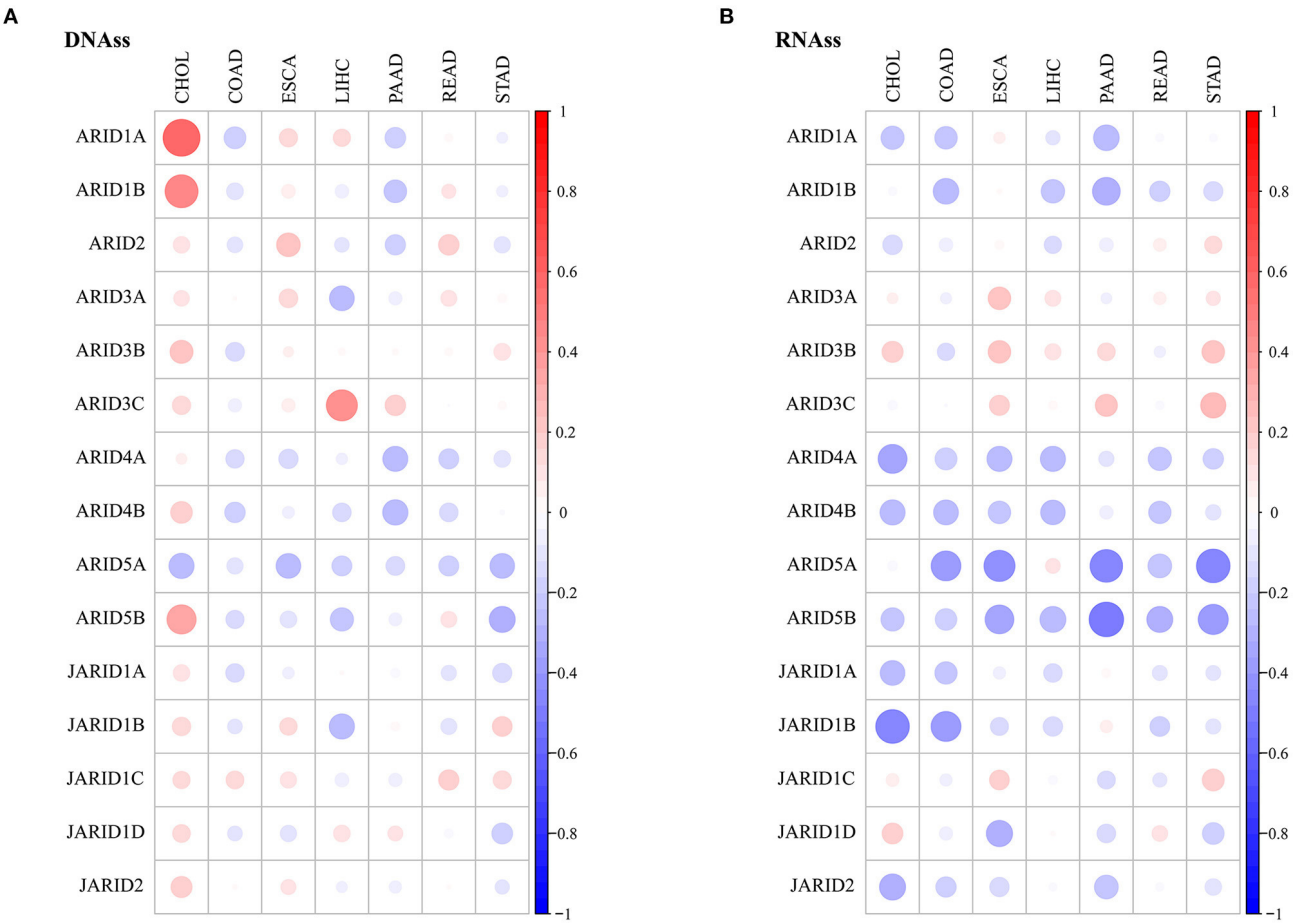
Association of ARIDs expressions with tumour stemness. Correlations between ARIDs expressions and DNAss **(A)** or RNAss **(B)** of digestive cancer. Bigger dot stands for higher correlation coefficient.

Tumour growth is fuelled by cancer stem cells, and cancer stemness is a critical characteristic that interferes with therapeutic effects. In our study, we used measured indicators to quantify cancer stemness, and the RNA stemness score (RNAss) and DNA stemness score (DNAss) were calculated based on mRNA expression and DNA methylation patterns. Our exploration showed that the correlation between the expression of the ARIDs and stemness was diverse in different types of digestive cancer (Figure 4; Supplementary Tables 4, 5). In most cancer types, specific genes were contrarily associated with RNAss and DNAss. *ARID1A* and *ARID1B* had a positive association with DNAss in CHOL, while the correlations were negative or insignificant with RNAss. These results suggest that RNAss and DNAss might identify stemness features from different aspects, and the independent evaluation of stemness is needed in digestive cancers. However, the expression of specific ARID genes was not consistent in every cancer. *ARID5A* was negatively associated with the DNAss in ESCA, LIHC and STAD and with the RNAss in COAD, ESCA, PAAD and READ.

### Expression of the ARID Gene Family Members in LIHC

**Figure 5:**
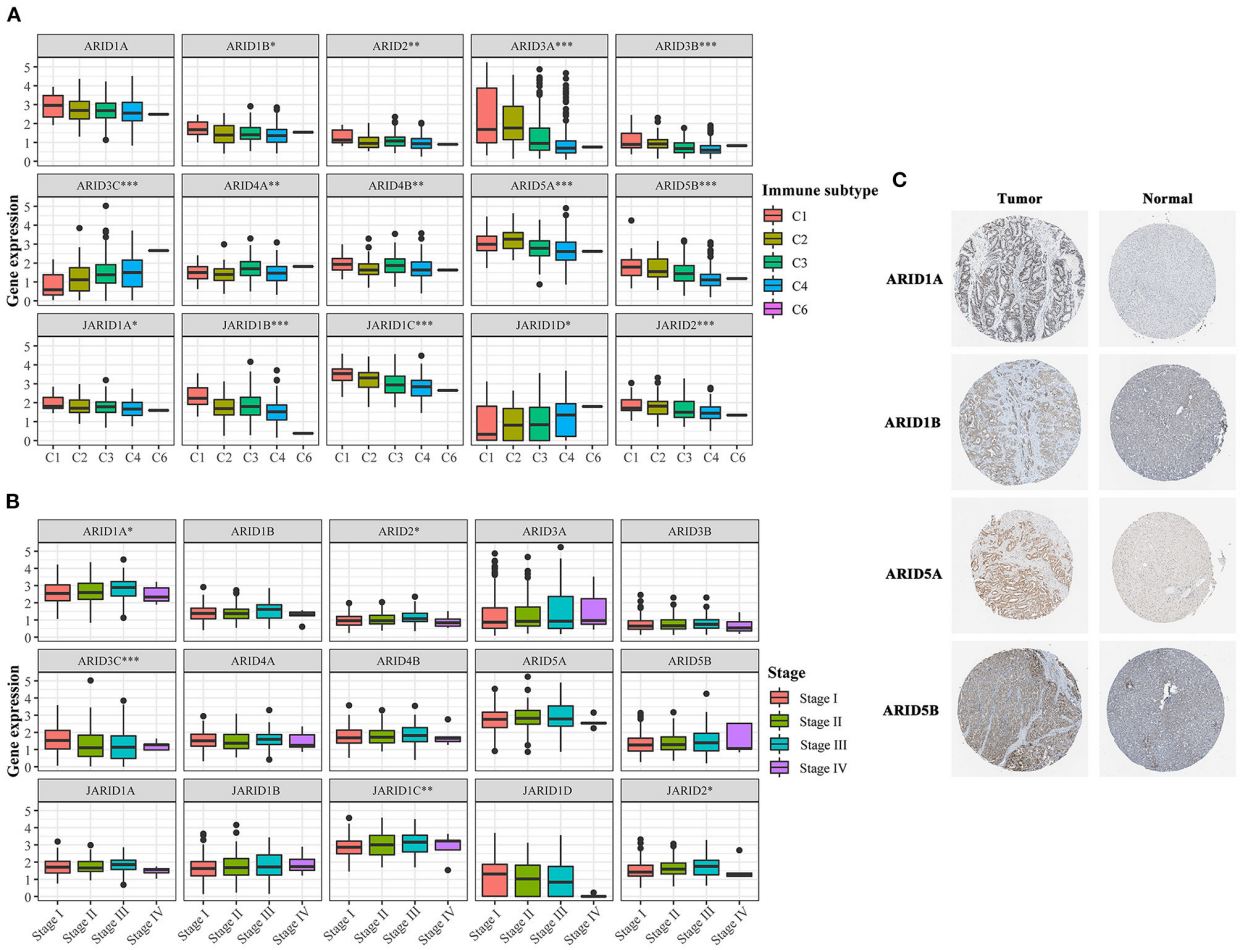
ARID family expressions in LIHC. **(A)** Association of ARIDs expression with immune infiltrate subtypes in LIHC. **(B)** Association of ARIDs expression with TNM stage in LIHC. **(C)** The protein level of the genes in HPA database. *P < 0.05, **P < 0.01, ***P < 0.001.

As shown in our results, the expression of ARIDs was tightly associated with LIHC patient survival. Here, we provide a thorough investigation of the expression of the ARIDs in a LIHC patient cohort from TCGA data. Except for *ARID1A*, the other 14 genes were significantly differentially expressed among immune subtypes (Figure 5A). The expression of *ARID3C* in different immune-infiltrated patterns varied by up to two-fold. The expressions of *ARID1A, ARID2, ARID3C, JARID1C* and *JARID2* were strongly correlated with tumour stage (Figure 5B). The expression of *JARID1C* was distinctly upregulated with increasing stage. Likewise, the protein expression levels of *ARID1A, ARID1B, ARID5A*, and *ARID5B* were significantly increased in LIHC (Figure 5C).

## Discussion

Previous studies have confirmed that the ARID gene family is highly associated with tumour growth in proliferation, apoptosis, migration, and invasion. As a conserved family, the expression and functions of these genes retain tightly interactive relationships. However, few studies have focused on the integral study of the 15 genes. Our study provides the first systemic analysis of ARID gene family members in the digestive system. We found noticeable heterogeneity in the expression levels of ARID genes among different tumours which suggested the works by these genes were not conservative. There was intersection among the functions of gene family in digestive cancers. However, the functions of different genes or in different cancers were still variant and the expression heterogenicity was an external manifestation. *ARID3C*, working as a transcriptional coactivator and a potential regulator of early processes in B cell antigen receptor signalling, was expressed preferentially in B lineage lymphocytes, and *ARID3A* had the highest expression levels in activated follicular B cells (24, 25). *ARID3C* had notable differential expression between tumour and normal tissues in CHOL, and our immune analysis showed that *ARID3C* expression was negatively associated with immune score in this type of cancer. Considering the role of *ARID3C* in lymphocytes, we inferred that it might be effectively activated through B cell antigen receptors to influence CHOL progression. It prompted the different regulator roles of ARIDs like activator or inhibitor in pathways were at work in the family heterogenicity among different types of digestive cancers.

*ARID1A* and *ARID1B* are two mutually exclusive regulatory subunits within the multiprotein chromatin remodelling complex SWI/SNF. Indeed, we found that they had similar expression patterns in most of the tested cancer types. Furthermore, they had similar expression patterns in immune infiltration subtypes, as they were highly expressed in immune subtypes enriched with IFN-γ and TGF-β infiltrates. Previous studies have confirmed that *ARID1A* deficiency leads to the loss of the tumour suppressive function of TGF-β (26). Mutations in *ARID1B* and TGF-β were simultaneously detected in liver metastases, but direct evidence of their association is still lacking in existing studies (27). *ARID2* could function as a tumour suppressor gene in colorectal cancer and constitute an important downstream element in the regulatory pathway of TGF-β in LIHC (28, 29). IFN-γ is a cytotoxic molecule secreted from immune cells and promotes immunomodulation and anticancer activity. A pronounced difference in PD-L1 induction by IFN-γ treatment existed between *ARID1A*-deficient and control gastric cancer cells (30). Downregulation of *ARID2* inhibited DNA methyltransferase, which increased methylation in the promoter region and increased the transcription of IFN-γ (31). Therefore, similar downstream mechanisms of ARIDs might share common characteristics in tumour regulation, and we could utilise these findings for the further investigation of other genes in digestive cancer.

The RNAss and DNAss that we referred to in this study were calculated based on machine learning which reflected the relationships between expression and stemness respectively from transcriptional and epigenetic regulation which might not be fully coincident. Strong correlation indicated family gene meddled the stemness of digestive cancer. Positive association theoretically contributed to the cancer stem cell and the negative promoted dormancy. The clinical value of *ARID1A* and *ARID1B* was profound in CHOL patients (32). Interestingly, although *ARID1A* and *ARID1B* had a positive association with DNAss estimated to have epigenetic modifications in CHOL, these two genes showed either a negative correlation or a correlation that was not significant with stemness when measured by RNAss. Our results indicated that *ARID1A* and *ARID1B* could alter the biological behaviour of CHOL cells through epigenetic regulation of cancer stemness. The expression of *ARID1A* impacted the biological functions, including migration, invasion and sphere formation activity, of CHOL and was confirmed to exhibit a tumour-suppressive effect through the regulation of *ALDH1A1*, a stemness marker, with histone acetylation (33). The role of *ARID1B* in stem cell-like features was unclear in previous CHOL studies. However, evidence supports that *ARID1A* and *ARID1B* work together in DNA modulation, which might provide a basis for subsequent investigations of stemness regulation (34). On these grounds, the associations with stemness might not be direct intervention.

The expression of 6 of the 15 gene family members was associated with the prognosis of LIHC patients, and the results were coincident with the hazard regression analysis. We chose the LIHC transcriptome for further analysis. Except for prognostic significance, most of the family members were associated with the immune subtype and cancer stage, indicating that these genes play roles in tumour progression and immune microenvironment abnormalities. Bioinformatics analysis of public databases and expression analysis of LIHC tissue samples showed that *ARID4B* was highly expressed in LIHC compared with adjacent normal tissues and strongly correlated with clinical features (35). The expression of *ARID1A* and *ARID2* inhibited the proliferation and migration of LIHC cells and, thus, act as tumour suppressors (36, 37). Therefore, the predicted roles of the ARID gene family members as a tumour suppressor or promoter in LIHC were in accord with our results, but validation from further experiments is required for an in-depth exploration.

The expression signature presented in our study might still have some limitations that may need further improvement. First, we performed an analysis based on transcriptome data from public databases, and more clinical trials should be included for confirmation in larger populations. Second, biological experiments and in-depth investigations of the potential mechanisms for the ARID gene family members in our study may better support the signature. Third, we selected digestive cancer for research because almost every ARID gene is related to several tumours in these organs and the internal heterogeneity among specific systems is less than that among whole organs. However, studies of ARIDs in pan-cancer may have their own advantages in further expanding this research.

We provided a comprehensive and systematic study of the expression of all ARID gene family members in patients with digestive cancer. Our results indicated that the putative tumour promoter or suppressor role of ARIDs was not consistent among the gene family members within a specific cancer type and that even the specific ARID member behaved differently in single or various cancer types. Taken together, our work uncovered the roles of the ARID gene family members in tumorigenesis, including the immune response and tumour microenvironment of digestive cancer, and might help to develop personalised treatment.

## Data Availability Statement

The original contributions presented in the study are included in the article/Supplementary Material, further inquiries can be directed to the corresponding authors.

## Author Contributions

WF had designed the study. YL and XZ had collected data. ZL, WW, and XC had analysed and interpreted the data. All authors were involved in writing paper and approved of the submitted and published versions.

## Funding

This work was supported by grants from National Natural Science Foundation of China (Nos. 91959110 and 81972702), Natural Science Foundation of Beijing (No. 7204324) and National Multidisciplinary Cooperative Diagnosis and Treatment Capacity building project for major diseases: comprehensive diagnosis and treatment of gastrointestinal tumours.

## Conflict of Interest

The authors declare that the research was conducted in the absence of any commercial or financial relationships that could be construed as a potential conflict of interest.

## Publisher's Note

All claims expressed in this article are solely those of the authors and do not necessarily represent those of their affiliated organizations, or those of the publisher, the editors and the reviewers. Any product that may be evaluated in this article, or claim that may be made by its manufacturer, is not guaranteed or endorsed by the publisher.
