# Supplementary material for: Expression Signature of the AT-Rich Interactive Domain Gene Family Identified in Digestive Cancer
Source: Front Med (Lausanne). 2022 Jan 20;8:775357. doi: 10.3389/fmed.2021.775357 (PMC8811461; doi:10.3389/fmed.2021.775357)
Supplement: Supplementary file 1 [file Data_Sheet_1.docx]

**Supplementary Table 1.** Summary of included cancer data

| Cancer type | Patients (n) | Tumor samples (n) | Normal samples (n) |
| --- | --- | --- | --- |
| CHOL | 33 | 36 | 9 |
| COAD | 439 | 471 | 41 |
| ESCA | 157 | 162 | 11 |
| LIHC | 346 | 374 | 50 |
| PAAD | 172 | 178 | 4 |
| READ | 158 | 167 | 10 |
| STAD | 337 | 376 | 31 |

**Supplementary Table 2.** Univariate Cox proportional hazard regression for prognosis analysis

| Family member | Cancer type | HR | 95% CI | | P-value |  |
| --- | --- | --- | --- | --- | --- | --- |
| *ARID1A* | CHOL | 0.135726 | 0.006356 | 2.898376 | 0.201022 |  |
|  | COAD | 0.783676 | 0.305291 | 2.011677 | 0.612305 |  |
|  | ESCA | 0.2997 | 0.096026 | 0.935376 | 0.037986 |  |
|  | LIHC | 1.061533 | 0.621135 | 1.814184 | 0.827128 |  |
|  | PAAD | 2.130339 | 0.651212 | 6.969072 | 0.211059 |  |
|  | READ | 1.923371 | 0.191059 | 19.36237 | 0.578795 |  |
|  | STAD | 0.956311 | 0.471952 | 1.937765 | 0.901332 |  |
| *ARID1B* | CHOL | 0.567704 | 0.01157 | 27.8564 | 0.775629 |  |
|  | COAD | 0.953493 | 0.212342 | 4.28153 | 0.950446 |  |
|  | ESCA | 0.070109 | 0.009797 | 0.501714 | 0.008124 |  |
|  | LIHC | 0.806252 | 0.25632 | 2.536053 | 0.712626 |  |
|  | PAAD | 3.207534 | 0.613899 | 16.75891 | 0.167101 |  |
|  | READ | 0.519507 | 0.015489 | 17.42494 | 0.714821 |  |
|  | STAD | 1.239708 | 0.450482 | 3.411621 | 0.677391 |  |
| *ARID2* | CHOL | 0.161735 | 0.008137 | 3.214736 | 0.232329 |  |
|  | COAD | 0.468031 | 0.130591 | 1.677394 | 0.243712 |  |
|  | ESCA | 0.580973 | 0.125578 | 2.687812 | 0.487148 |  |
|  | LIHC | 0.813703 | 0.262445 | 2.522866 | 0.721026 |  |
|  | PAAD | 3.980099 | 0.772372 | 20.5098 | 0.098696 |  |
|  | READ | 0.464829 | 0.015221 | 14.19508 | 0.660542 |  |
|  | STAD | 1.148707 | 0.36477 | 3.617418 | 0.812755 |  |
| *ARID3A* | CHOL | 0.820743 | 0.074035 | 9.098642 | 0.872136 |  |
|  | COAD | 0.968907 | 0.491239 | 1.911047 | 0.927377 |  |
|  | ESCA | 1.459138 | 0.688126 | 3.09403 | 0.324487 |  |
|  | LIHC | 1.260582 | 0.872146 | 1.82202 | 0.217907 |  |
|  | PAAD | 0.126942 | 0.032833 | 0.490795 | 0.002776 |  |
|  | READ | 1.194251 | 0.313584 | 4.54817 | 0.794715 |  |
|  | STAD | 1.058165 | 0.670739 | 1.669371 | 0.807968 |  |
| *ARID3B* | CHOL | 0.32213 | 0.017757 | 5.843932 | 0.443631 |  |
|  | COAD | 2.589456 | 0.5858 | 11.44638 | 0.209579 |  |
|  | ESCA | 0.972122 | 0.26831 | 3.522129 | 0.965664 |  |
|  | LIHC | 0.857274 | 0.282016 | 2.605946 | 0.786021 |  |
|  | PAAD | 0.537669 | 0.114819 | 2.51778 | 0.43085 |  |
|  | READ | 1.320051 | 0.047216 | 36.90598 | 0.870207 |  |
|  | STAD | 0.7474 | 0.313179 | 1.783663 | 0.511788 |  |
| *ARID3C* | CHOL | 6.078537 | 0.103337 | 357.555 | 0.385316 |  |
|  | COAD | 1.985339 | 0.14021 | 28.11196 | 0.612057 |  |
|  | ESCA | 0.748292 | 0.000551 | 1016.075 | 0.937205 |  |
|  | LIHC | 0.725372 | 0.493732 | 1.065688 | 0.101877 |  |
|  | PAAD | 0.002407 | 1.65E-06 | 3.51517 | 0.104843 |  |
|  | READ | 34.84553 | 0.951443 | 1276.178 | 0.053252 |  |
|  | STAD | 0.325173 | 0.016476 | 6.417543 | 0.460355 |  |
| *ARID4A* | CHOL | 0.198391 | 0.020345 | 1.934581 | 0.163906 |  |
|  | COAD | 1.155771 | 0.336408 | 3.970797 | 0.818171 |  |
|  | ESCA | 0.674601 | 0.192043 | 2.36971 | 0.539175 |  |
|  | LIHC | 0.545413 | 0.263458 | 1.129115 | 0.102496 |  |
|  | PAAD | 3.232952 | 0.749762 | 13.94039 | 0.115554 |  |
|  | READ | 2.20722 | 0.154137 | 31.60709 | 0.559886 |  |
|  | STAD | 1.187062 | 0.51264 | 2.748746 | 0.688952 |  |
| *ARID4B* | CHOL | 0.411683 | 0.039356 | 4.306397 | 0.45872 |  |
|  | COAD | 0.881808 | 0.289152 | 2.68919 | 0.825018 |  |
|  | ESCA | 0.679164 | 0.192458 | 2.396696 | 0.547606 |  |
|  | LIHC | 0.606353 | 0.280153 | 1.312364 | 0.204103 |  |
|  | PAAD | 5.267673 | 1.273434 | 21.79019 | 0.021812 |  |
|  | READ | 4.186412 | 0.30062 | 58.29974 | 0.286633 |  |
|  | STAD | 1.25149 | 0.533182 | 2.937509 | 0.606327 |  |
| *ARID5A* | CHOL | 0.501961 | 0.119794 | 2.103313 | 0.345755 |  |
|  | COAD | 1.554017 | 0.628509 | 3.84238 | 0.339843 |  |
|  | ESCA | 0.57106 | 0.215852 | 1.510802 | 0.259034 |  |
|  | LIHC | 0.800715 | 0.544896 | 1.176636 | 0.257761 |  |
|  | PAAD | 0.836101 | 0.370585 | 1.88638 | 0.666329 |  |
|  | READ | 10.01465 | 2.03257 | 49.34304 | 0.00463 |  |
|  | STAD | 1.461638 | 0.764646 | 2.793953 | 0.250886 |  |
| *ARID5B* | CHOL | 0.530613 | 0.136937 | 2.056062 | 0.359149 |  |
|  | COAD | 0.732863 | 0.311143 | 1.726181 | 0.477062 |  |
|  | ESCA | 0.537944 | 0.236622 | 1.222978 | 0.13898 |  |
|  | LIHC | 1.03058 | 0.527501 | 2.013447 | 0.929756 |  |
|  | PAAD | 2.003851 | 0.831221 | 4.830747 | 0.121573 |  |
|  | READ | 1.827322 | 0.235232 | 14.19498 | 0.564369 |  |
|  | STAD | 1.42873 | 0.760065 | 2.685653 | 0.267871 |  |
| *JARID1A* | CHOL | 0.131097 | 0.01339 | 1.283495 | 0.08089 |  |
|  | COAD | 1.06913 | 0.360313 | 3.172355 | 0.904119 |  |
|  | ESCA | 0.462593 | 0.106066 | 2.017538 | 0.304932 |  |
|  | LIHC | 0.483093 | 0.191598 | 1.218062 | 0.123097 |  |
|  | PAAD | 2.286409 | 1.137749 | 4.594742 | 0.020213 |  |
|  | READ | 0.528922 | 0.080269 | 3.485239 | 0.507917 |  |
|  | STAD | 1.464897 | 0.581464 | 3.690553 | 0.418032 |  |
| *JARID1B* | CHOL | 0.616053 | 0.116952 | 3.245107 | 0.567717 |  |
|  | COAD | 0.902821 | 0.360442 | 2.26135 | 0.827257 |  |
|  | ESCA | 0.511647 | 0.172505 | 1.517541 | 0.227026 |  |
|  | LIHC | 1.42063 | 0.795691 | 2.536397 | 0.235155 |  |
|  | PAAD | 7.222649 | 2.539812 | 20.53957 | 0.000209 |  |
|  | READ | 1.301929 | 0.183351 | 9.244646 | 0.791922 |  |
|  | STAD | 1.611419 | 0.782163 | 3.31986 | 0.195753 |  |
| *JARID1C* | CHOL | 0.388975 | 0.047173 | 3.207387 | 0.380365 |  |
|  | COAD | 1.446025 | 0.584253 | 3.578905 | 0.425068 |  |
|  | ESCA | 0.434424 | 0.112974 | 1.670511 | 0.225032 |  |
|  | LIHC | 0.983485 | 0.547833 | 1.76558 | 0.955518 |  |
|  | PAAD | 1.366426 | 0.508445 | 3.672217 | 0.535945 |  |
|  | READ | 3.180219 | 0.508219 | 19.90048 | 0.216254 |  |
|  | STAD | 0.620413 | 0.301336 | 1.277351 | 0.195114 |  |
| *JARID1D* | CHOL | 2.080884 | 0.718968 | 6.022626 | 0.176545 |  |
|  | COAD | 0.887171 | 0.496421 | 1.585494 | 0.686118 |  |
|  | ESCA | 0.751692 | 0.374031 | 1.510675 | 0.422846 |  |
|  | LIHC | 0.563734 | 0.366094 | 0.868073 | 0.00926 |  |
|  | PAAD | 0.810333 | 0.500029 | 1.313203 | 0.393212 |  |
|  | READ | 0.585472 | 0.192542 | 1.780273 | 0.345438 |  |
|  | STAD | 1.475201 | 0.961483 | 2.263396 | 0.075055 |  |
| *JARID2* | CHOL | 2.179193 | 0.074085 | 64.10035 | 0.651634 |  |
|  | COAD | 0.940398 | 0.270476 | 3.269599 | 0.923 |  |
|  | ESCA | 0.502303 | 0.179267 | 1.407447 | 0.19026 |  |
|  | LIHC | 0.885034 | 0.393631 | 1.989893 | 0.767657 |  |
|  | PAAD | 2.827252 | 0.536843 | 14.88956 | 0.220158 |  |
|  | READ | 0.524578 | 0.026044 | 10.56607 | 0.67368 |  |
|  | STAD | 1.461764 | 0.673612 | 3.172084 | 0.336838 |  |

**Supplementary Table 3.** P-value of ESTIMATE score

| Family member | CHOL | COAD | ESCA | LIHC | PAAD | READ | STAD |
| --- | --- | --- | --- | --- | --- | --- | --- |
| *ARID1A* | 0.110189 | 0.006454 | 0.048208 | 0.019679 | 0.000297 | 0.201556 | 0.86448 |
| *ARID1B* | 0.138752 | 0.21147 | 0.811976 | 0.44237 | 4.38E-08 | 0.264373 | 0.207234 |
| *ARID2* | 0.776424 | 0.076248 | 0.53893 | 0.017425 | 0.235465 | 0.077246 | 0.963535 |
| *ARID3A* | 0.20843 | 3.45E-07 | 0.05237 | 0.264577 | 0.976183 | 0.100913 | 0.250077 |
| *ARID3B* | 0.460254 | 0.9043 | 0.275436 | 0.384588 | 0.338991 | 0.804473 | 0.172702 |
| *ARID3C* | 0.226165 | 0.058371 | 0.070437 | 0.007507 | 0.000156 | 0.164569 | 7.55E-05 |
| *ARID4A* | 0.924217 | 0.00445 | 0.000367 | 0.207737 | 0.002628 | 0.197928 | 0.322929 |
| *ARID4B* | 0.543763 | 0.002286 | 0.041849 | 0.021691 | 0.016734 | 0.3372 | 0.023499 |
| *ARID5A* | 0.001286 | 4.37E-11 | 0 | 0.000686 | 0 | 0.007221 | 0 |
| *ARID5B* | 0.094417 | 2.67E-12 | 5.75E-06 | 1.01E-09 | 0 | 0.00321 | 0 |
| *JARID1A* | 0.593797 | 0.000264 | 0.770209 | 0.32464 | 0.036683 | 0.569428 | 0.006987 |
| *JARID1B* | 0.666402 | 0.195077 | 0.005583 | 0.996019 | 0.00833 | 0.661817 | 3.53E-06 |
| *JARID1C* | 0.893341 | 0.005652 | 0.137687 | 0.147216 | 0.637434 | 0.055675 | 0.06293 |
| *JARID1D* | 0.156237 | 0.778274 | 0.000621 | 0.615095 | 0.924376 | 0.701244 | 0.032037 |
| *JARID2* | 0.121396 | 0.804027 | 0.83743 | 0.110326 | 3.38E-08 | 0.203666 | 0.132573 |

**Supplementary Table 4.** P-value of DNAss

| Family member | CHOL | COAD | ESCA | LIHC | PAAD | READ | STAD |
| --- | --- | --- | --- | --- | --- | --- | --- |
| *ARID1A* | 0.000352 | 0.000696 | 0.084011 | 0.018455 | 0.026598 | 0.810522 | 0.385761 |
| *ARID1B* | 0.005905 | 0.050013 | 0.337408 | 0.150873 | 0.007729 | 0.438852 | 0.349028 |
| *ARID2* | 0.510282 | 0.087486 | 0.003341 | 0.093099 | 0.033352 | 0.093006 | 0.045896 |
| *ARID3A* | 0.561905 | 0.938684 | 0.069228 | 7.74E-07 | 0.385472 | 0.319067 | 0.561795 |
| *ARID3B* | 0.198048 | 0.012881 | 0.601811 | 0.716077 | 0.865721 | 0.825485 | 0.030852 |
| *ARID3C* | 0.419382 | 0.221058 | 0.358897 | 0 | 0.030446 | 0.992115 | 0.629883 |
| *ARID4A* | 0.789096 | 0.018129 | 0.052144 | 0.310071 | 0.001085 | 0.11233 | 0.034801 |
| *ARID4B* | 0.254383 | 0.003188 | 0.468416 | 0.006195 | 0.000815 | 0.169225 | 0.876404 |
| *ARID5A* | 0.122147 | 0.065794 | 0.001352 | 0.001593 | 0.069245 | 0.111032 | 2.89E-06 |
| *ARID5B* | 0.034278 | 0.020923 | 0.146665 | 1.44E-05 | 0.410909 | 0.314219 | 1.67E-07 |
| *JARID1A* | 0.512222 | 0.017344 | 0.495643 | 0.911226 | 0.666761 | 0.36178 | 0.005246 |
| *JARID1B* | 0.412142 | 0.136949 | 0.116485 | 3.24E-07 | 0.695847 | 0.310281 | 0.003079 |
| *JARID1C* | 0.473205 | 0.026307 | 0.17018 | 0.13387 | 0.373911 | 0.101676 | 0.011721 |
| *JARID1D* | 0.450897 | 0.131571 | 0.170342 | 0.027248 | 0.258578 | 0.765171 | 0.000908 |
| *JARID2* | 0.280758 | 0.892465 | 0.242724 | 0.349982 | 0.445984 | 0.962334 | 0.128499 |

**Supplementary Table 5.** P-value of RNAss

| Family member | CHOL | COAD | ESCA | LIHC | PAAD | READ | STAD |
| --- | --- | --- | --- | --- | --- | --- | --- |
| *ARID1A* | 0.189594 | 8.64E-06 | 0.513418 | 0.108124 | 0.000582 | 0.749676 | 0.652426 |
| *ARID1B* | 0.873234 | 7.19E-09 | 0.91795 | 1.06E-05 | 5.10E-05 | 0.032718 | 0.003526 |
| *ARID2* | 0.356656 | 0.113687 | 0.685059 | 0.01831 | 0.329775 | 0.402749 | 0.01972 |
| *ARID3A* | 0.791406 | 0.297145 | 0.007555 | 0.045943 | 0.560982 | 0.429053 | 0.117202 |
| *ARID3B* | 0.280758 | 0.009226 | 0.00769 | 0.03924 | 0.109272 | 0.51957 | 5.24E-05 |
| *ARID3C* | 0.84161 | 0.959327 | 0.036541 | 0.553341 | 0.011986 | 0.725932 | 3.22E-07 |
| *ARID4A* | 0.038154 | 2.40E-05 | 0.000876 | 3.45E-07 | 0.23656 | 0.005056 | 0.000894 |
| *ARID4B* | 0.122147 | 5.35E-08 | 0.008091 | 1.30E-06 | 0.36725 | 0.009816 | 0.065105 |
| *ARID5A* | 0.827425 | 9.33E-16 | 1.61E-07 | 0.088583 | 9.48E-09 | 0.003423 | 0 |
| *ARID5B* | 0.189074 | 4.55E-05 | 8.99E-06 | 5.00E-08 | 3.29E-11 | 0.000419 | 1.30E-13 |
| *JARID1A* | 0.13506 | 1.29E-05 | 0.446975 | 0.006787 | 0.687399 | 0.252694 | 0.086503 |
| *JARID1B* | 0.003904 | 3.37E-16 | 0.092395 | 0.002651 | 0.445305 | 0.044564 | 0.073965 |
| *JARID1C* | 0.696141 | 0.211312 | 0.042392 | 0.548713 | 0.101097 | 0.313465 | 0.000141 |
| *JARID1D* | 0.298365 | 0.137169 | 0.000262 | 0.882277 | 0.076205 | 0.209253 | 0.000188 |
| *JARID2* | 0.093493 | 0.000309 | 0.051537 | 0.633153 | 0.003267 | 0.738134 | 0.030814 |
